# Interoception and Autonomic Correlates during Social Interactions. Implications for Anorexia

**DOI:** 10.3389/fnhum.2017.00219

**Published:** 2017-05-17

**Authors:** Marianna Ambrosecchia, Martina Ardizzi, Elisa Russo, Francesca Ditaranto, Maurizio Speciale, Piergiuseppe Vinai, Patrizia Todisco, Sandra Maestro, Vittorio Gallese

**Affiliations:** ^1^Department of Medicine and Surgery, Unit of Neuroscience, University of ParmaParma, Italy; ^2^Casa di Cura, Villa MargheritaVicenza, Italy; ^3^Istituto di Ricovero e Cura a Carattere Scientifico (IRCCS), Fondazione Stella MarisPisa, Italy; ^4^“GNOSIS” Research and Psychotherapy GroupMondovì, Italy; ^5^Institute of Philosophy, School of Advanced Study, University of LondonLondon, UK

**Keywords:** anorexia nervosa, autonomic reactivity, bodily self, interoception, interoceptive accuracy, proxemics, sinus respiratory arrhythmia, social interaction

## Abstract

The aim of this study is to investigate the bodily-self in Restrictive Anorexia, focusing on two basic aspects related to the bodily self: autonomic strategies in social behavior, in which others' social desirability features, and social cues (e.g., gaze) are modulated, and interoception (i.e., the sensitivity to stimuli originating inside the body). Furthermore, since previous studies carried out on healthy individuals found that interoception seems to contribute to the autonomic regulation of social behavior, as measured by Respiratory Sinus Arrhythmia (RSA), we aimed to explore this link in anorexia patients, whose ability to perceive their bodily signal seems to be impaired. To this purpose, we compared a group of anorexia patients (ANg; restrictive type) with a group of Healthy Controls (HCg) for RSA responses during both a resting state and a social proxemics task, for their explicit judgments of comfort in social distances during a behavioral proxemics task, and for their Interoceptive Accuracy (IA). The results showed that ANg displayed significantly lower social disposition and a flattened autonomic reactivity during the proxemics task, irrespective of the presence of others' socially desirable features or social cues. Moreover, unlike HCg, the autonomic arousal of ANg did not guide behavioral judgments of social distances. Finally, IA was strictly related to social disposition in both groups, but with opposite trends in ANg. We conclude that autonomic imbalance and its altered relationship with interoception might have a crucial role in anorexia disturbances.

## Introduction

“How much, how much I enjoy the streamlinedness of it, the simplicity. I really care about that. But I couldn't stay alive. My “less is more” sort of thing, and also wishing to feel the consciousness of my body. So the coupling of a variety of things made me arrive at this very, very streamlined diet in which there clearly wasn't sufficient nutrition to sustain life” (Bruch, 1973).

“Sometimes I feel if I'm made of glass, like I'm transparent, and everyone can see right into my side, it makes me want to scream, get out, get out of me!” (Recovering anorexic personal communication, in Lester, 1997).

Anorexia nervosa (AN) is an eating disorder characterized by restriction of food energy intake due to an irrational fear of gaining weight and a distorted way in which body shape and weight are experienced that have an inappropriate influence on self- evaluation (DSM V—American Psychiatric Association, 2013). The serious loss of weight leads to severe malnutrition and an alarming high mortality risk compared with other psychiatric illnesses (Sullivan, 1995; Casiero and Frishman, 2006). In the last decades, the frequency of this illness and other eating disorders greatly increased (Fassino et al., 2004; Friederich et al., 2006; Keski-Rahkonen et al., 2007). Anorexia significantly impact health care, mostly in the female population, and represents a great challenge for physicians of various specialties (Mitchell and Crow, 2006).

To date, the etiology of this illness remains not yet fully understood. There is also increasing need for developing more effective treatments (Herzog et al., 1992; Vandereycken, 2003; Jacobi et al., 2004; Fairburn and Bohn, 2005; Tchanturia et al., 2005; Riva, 2014). In the last years, thanks to the support of neuroscience, several neurobiological models of eating disorders emerged; Kaye et al. (2009, 2010, 2013, 2015) for example, consider AN as the product of an altered serotonin and dopamine metabolism which in turn may leads to dysfunctional neural process involved in emotion and appetite. Such alterations would contribute to AN trait-related vulnerabilities like anxiety, emotional recognition and regulation deficits (Schmidt et al., 1993; Zonnevijlle-Bendek et al., 2002; Kucharska-Pietura et al., 2004; Schmidt and Treasure, 2006; Harrison et al., 2009; Rowsell et al., 2016), insensitivity to reward (Kaye et al., 2009; Harrison et al., 2010), disturbed perception of physical states (Fassino et al., 2004; Pollatos et al., 2008; see below) and cognitive inflexibility and rigidity (Katzman et al., 2001; Anderluh et al., 2003; Kucharska-Pietura et al., 2004; Tchanturia et al., 2004; Cassin and von Ranson, 2005; Chui et al., 2008; Titova et al., 2013) that may be exacerbated by puberty and social desirability, given rise to the onset of AN.

In addition, Treasure and Schmidt (2013) and Schmidt and Treasure (2006) in their cognitive—interpersonal maintenance model of eating disorders identified cognitive, socio-emotional, and interpersonal elements whose joint action would be involved in causing and maintaining eating disorders. Specifically, they suggest that obsessive compulsive and anxious avoidant traits may encourage anorexia beliefs and behaviors, determining widely documented problems in interpersonal relationships (Kog and Vandereycken, 1989; Kucharska-Pietura et al., 2004; Russell et al., 2009; Oldershaw et al., 2010; Watson et al., 2010; Claes et al., 2012; Zucker et al., 2013). Finally, Fairburn et al. (2003) proposed the Trans diagnostic theory of eating disorder, highlighting the role of self-esteem, perfectionism, and mood intolerance as core factors of eating disorder maintenance.

However, even if these models importantly increased the knowledge about the underpinnings of eating disorders, they only partially addressed the role of bodily experience in this pathology. Nonetheless, as previously mentioned (see above), disturbances in the way in which body weight or shape are experienced represent core symptoms of AN, (DSM V—American Psychiatric Association, 2013), in which the body is refused, lived as an object from which to get away (Noll and Fredrickson, 1998; Daubenmier, 2005; Riva et al., 2015). This gap has been recently filled by the Allocentric Lock Theory (see Riva, 2014; Riva et al., 2014), which conceives of EDs as the outcome of impaired ability in updating a negative bodily representation stored in autobiographical memory (allocentric) with real-time sensorimotor and proprioceptive data (egocentric; Riva, 2014). In line with Embodied Social Cognition theories, these authors highlighted the central role of the physical body in influencing the mind. This perspective emphasizes the link between altered (physical) subjective experience and both disturbed inter-subjectivity and neurobiological dysfunctions in the development of the mental illness (Matthews, 2004, 2007; Ratcliffe, 2008; Fuchs and Schlimme, 2009; Glannon, 2009; Gallese and Ferri, 2013; Gallese, 2014).

An embodied view of AN is also supported by patients' experiences (see above), through which it is quite evident that this disorder may reflect something more than a mere body image disorder (i.e., perceptual overestimation of one's body appearance and cognitive-evaluative dissatisfaction and disparagement—Cash and Deagle, 1997). Indeed, AN looks like a struggle with deeper and low-level aspects of the self, involving more implicit and unconscious aspects of the bodily-self such as action-oriented body schema (Guardia et al., 2010, 2012; Nico et al., 2010; Keizer et al., 2013), interoception (Fassino et al., 2004; Pollatos et al., 2008; Herbert and Pollatos, 2012; Strigo et al., 2013), multisensory body perception (see Gaudio et al., 2014 for a review), multisensory integration (Eshkevari et al., 2012), influencing both the body image and AN behaviors. For example, Epstein et al. (2001) found that patients in acute phase of AN showed poorer proprioceptive abilities compared to controls. In addition, Nico et al. (2010) tested body size perception implicitly, carrying out a psychophysical task in which participants had to predict whether a light beam would have hit/missed their body. They found that AN patients, like patients with right parietal lobe lesions, were significantly less precise than controls and underestimated eccentricity of their left body boundary.

Concerning interoception, it represents a core component of bodily-self experience, because it consists of the sensitivity to visceral stimuli originating inside of the body (Craig, 2002). It is often concomitant with emotional responses (Critchley et al., 2004; Gaudio et al., 2014). A strict relationship between IA and social attitudes in the context of real social interactions was the target of a recent study, carried out on healthy individuals (Ferri et al., 2013). This study demonstrated that IA contributes to inter-individual differences concerning social disposition and interpersonal space representation, via recruitment of different adaptive autonomic response strategies. The authors assessed Respiratory Sinus Arrhythmia (RSA) in both social and a non-social task. In the social task, participants viewed a caress-like movement, performed by an experimenter's hand, at different distances from participants' hand. In the non-social task, the movement of a metal stick replaced the hand. RSA is one of the periodic components of heart rate variability (Berntson et al., 1997) directly resulting from the interaction between the cardiovascular and respiratory systems (Grossman and Taylor, 2007). RSA is an index of social disposition (Porges et al., 2013) and positive social functioning both in healthy (Graziano et al., 2007) and clinical samples (Bal et al., 2010; Patriquin et al., 2013), and it can be modulated by emotional processing (Porges and Smilen, 1994).

The results showed that only good heartbeat perceivers with high IA levels displayed stronger autonomic responses in the social setting compared to the non-social setting. Particularly, when the experimenter's hand was moving at the boundary of participants' peri-personal space (i.e., 20 cm from the participants' hand). On the contrary, poor heartbeat perceivers with low IA levels were less predisposed to social engagement, as they required more intrusive social stimuli to be delivered in their personal space (i.e., touching their hand) to effectively predispose the autonomic response to them (Ferri et al., 2013).

Interoception in eating disorders has been poorly assessed. Some authors found that individuals suffering from anorexia showed difficulty to discriminate not only visceral sensations related to eating behaviors, such as hunger and satiety (Garner et al., 1983; Fassino et al., 2004; Lilenfeld et al., 2006; Matsumoto et al., 2006), but also visceral sensation in general (Pollatos et al., 2008). A study by Pollatos et al. (2008), for example, found that anorexic patients showed lower Interoceptive Accuracy (IA; performance on objective behavioral tests about visceral sensation detection, see Garfinkel et al., 2015) in a well-assessed heartbeat detection task (Schandry, 1981). Coherently, these patients show altered activation of the anterior insula (Wagner et al., 2008; Oberndorfer et al., 2013), which seems to play a crucial role in interoception (Critchley et al., 2004; Pollatos et al., 2007; Craig, 2009). Anterior insula is also relevant for emotional processing (Phan et al., 2002), and for the self-regulation of feelings and behavior (Beauregard et al., 2001) and it has been recently proposed to be responsible for the altered disgust sensitivity in AN (e.g., Vicario, 2013; Moncrieff-Boyd et al., 2014; Hildebrandt et al., 2015).

Considering that AN seems to be associated to low levels of IA (Pollatos et al., 2008), together with a wide range of autonomic system disturbances whose nature is far from clear (for a review see Mazurak et al., 2011), and taking into account the demonstrated link between IA levels and autonomic regulation in social context among healthy individuals, the purpose of the present study was to assess AN patients' autonomic regulation in social contexts and its possible relation with IA.

To this aim, we assessed RSA and IA of both a group of AN patients (restrictive type) and a group of Healthy Controls. To test participants' autonomic reactivity during social interactions, the two groups were also submitted to a Physiological proxemics task, a modified version of the “personal space regulation task” used by Kennedy et al. (2009). During the task, participants were instructed to view, one by one, two female experimenters (the one obese, the other underweight) slowly approaching them, from a distance of 470 cm across the room to a tip-to-tip distance (about 30 cm), or vice versa, slowly moving away from participants. We recruited two experimenters with different BMI to test its possible influence on participants' responses. Furthermore, to explore the role of social cues in modulating participants' responses during social interaction, the presence or the absence of eye contact (from the experimenter toward the participant) were also introduced. Participants' electrocardiogram (ECG) was recorded (to extract RSA) for the entire duration of the Physiological proxemics task.

As an explicit measure of participants' comfort during social interaction, and to help us with the interpretation of physiological results, they were also submitted to a Behavioral proxemics task, that is, the behavioral version of the Physiological proxemics task without ECG recordings. In this task, participants had to explicitly stop the experimenter as soon as she reached a distance at which they felt most comfortable (closer could be too much and farer could be too less).

IA was assessed throughout a well-assessed heartbeat perception task, the same used by Pollatos et al. (2008), following the Mental tracking Method by Schandry (1981).

On the basis of previous studies, we hypothesized lower resting RSA responses and IA in ANg compared to HCg. We also hypothesized a compromised relationship between IA and social disposition in AN. Given that this is a relatively uncharted territory, we explored both within and between group differences in autonomic and behavioral reactivity in the different social context, where the interacting experimenters' eye contact and BMI were manipulated.

## Materials and method

### Participants

Twenty-four right-handed women diagnosed of Anorexia Nervosa, restrictive subtype, according to the DSM V criteria (American Psychiatric Association, 2013; AN group-ANg; mean age: 23.04 SE = 1.9; mean BMI: 16.1 Kg/m2 SE = 0.3; mean duration of illness: 6 years SE = 1.6; all females) were included in the study. The restrictive subtype of AN is characterized by the absence, during the last 3 months, of recurrent episodes of binge eating or purging behaviors as self-induced vomiting or the misuse of laxatives, diuretics, or enemas. All patients followed a controlled diet for the 10 days prior to the experiment in order to avoid the confounding effects of malnutrition on the performance.

Twenty-five control participants (HC group -HCg; mean age: 22.9 SE = 1.1; mean BMI: 21 Kg/m^2^ SE = 0.58; all females) with normal Body Mass Index (BMI comprised between 18.5 and 24.9) were matched with AN patients for age and gender. Exclusion criteria for both groups included actual or past cognitive disorders (mental retardation), psychiatric disorders (psychosis), severe medical illnesses (head trauma, neurological, and cardio-respiratory diseases, and diabetes), substance dependence, and intake of medications altering the cardio-respiratory activity. Given the frequent comorbidity in anorexia nervosa with major depression, anxiety, and personality disorders, these were not comprised among exclusion criteria for ANg, but they were carefully clinically assessed (see below). Furthermore, since it is known that the autonomic tone, especially the vagal component (de Geus et al., 1995; Jurca et al., 2004), is affected by regular exercise, improving, in turn, IA as assessed by heartbeat detection (Bestler et al., 1990; Herbert et al., 2010), only individuals not regularly involved in athletic or endurance sports were recruited. A further exclusion criterion for the control group was a personal history of eating disorders, and a clinical risk to develop an eating disorder (high risk score in BSQ, EDE-Q, and EDRC scale of EDI3).

In a previous and separate session from the experiment, all participants filled in several questionnaires including an anamnestic questionnaire, the Eating Disorder Inventory (EDI-3; Giannini et al., 2008) and the Eating Disorder Examination Questionnaire (EDE Q; Fairburn, 2008), to assess both the eating disorder risk and the symptomatology associated with eating disorders, the Body Uneasiness Test (BUT; Cuzzolaro et al., 2006) and the Body Shape Questionnaire (BSQ; Stefanile et al., 2011), to measure concerns about body shape. In addition, participants were required to filled in the Symptom Checklist-90 (SCL-90; Derogatis et al., 1973), to assess their current psychological status and to exclude psychopathological symptoms in HC. They also filled in the Dissociative Experiences Scale (DES; Carlson and Putnam, 1993) to explore their possible dissociative symptoms.

Since there is evidence suggesting that depression symptoms and RSA interact (Yaroslavsky et al., 2013, 2014), participants were also required to fill in the Italian version of the Beck's Depression Inventory (BDI; Ghisi et al., 2006). The BDI is a widely used 21-items multiple-choice self-report inventory that measures the presence and severity of affective, cognitive, motivational, psychomotor, and vegetative symptoms of depression.

Similarly, because it has been shown that anxiety interacts with RSA (Gorka et al., 2013; Mathewson et al., 2013) and there is evidence suggesting positive association between IA and anxiety (Van der Does et al., 2000; Pollatos et al., 2007, 2009), participants filled in the Italian version of the State-Trait Anxiety Inventory (Pedrabissi and Santinello, 1989). The STAI is a 40 items scale, which assesses both state (this latter was administered during the experimental session) and trait anxiety. It represents widely-validated and reliable self-report measures of trait and state anxiety.

Sociodemographic features and questionnaire scores obtained from the two groups of participants are shown in Table 1.

**Table 1:** **Comparison between the two groups with respect to socio-demographic and questionnaire data**.

	**ANg mean (SE)**	**HCg mean (SE)**	***T*(*df*=1,47)**	***p***
N(sex)	24 (f)	25 (f)	n.a.	n.a
Age	23 ± 9 (2)	23 ± 5.5 (1)	−0.7	n.s.
Illness duration, year	6 ± 8 (1.6)	n.a.	n.a.	n.a.
BMI	16 (0.3)	21 (0.6)	7.7	^***^
Weight	43.2 (0.8)	57 (1.9)	6.7	^***^
Height	1.6 (0.01)	1.6 (0.01)	0.18	n.s.
DES	20.5 (3.5)	9.1 (1.7)	−3	^***^
EDI 3-ID	77 (5.3)	32.8 (5.9)	−5.6	^***^
EDI3-LSE	81.9 (4.2)	30 (4.9)	−7.9	^***^
EDI3-II	71. (5.3)	42.2 (5.9)	−3.6	^**^
EDI3-ED	65.4 (5.9)	30.8 (5.6)	−3.9	^***^
EDI3-EDRC	74.2 (19.4)	27.9 (3.9)	−8.3	^***^
SCL-90	1.3 (0.12)	0.5(0.8)	−5.8	^***^
STAI- State	49.8 (2.2)	35(2.7)	−4.7	^***^
STAI- Trait	61.3 (2.2)	40.3 (2.2)	−6.8	^***^
BDI	27.2 (2.7)	6.5 (1.3)	−7	^***^
BUT (GSI)	2 (1.1)	0.8 (0.1)	−5	^***^
BUT (BIC)	1.9 (0.2)	0.9 (0.1)	−3.6	^***^
BSQ	121.3 (8.2)	56.3 (4)	−7.2	^***^

** *p < 01*,

*** *p < 001, n.s, not significant; n.a, not applicable*.

The experimental protocol was approved by the Ethics Committee of Casa di Cura Villa Margherita, Arcugnano, Vicenza, Italy. The experiment was conducted in accordance with the ethical standards of the 2013 Declaration of Helsinki and all participants involved in the study gave written informed consent.

### Procedure

Participants were required to abstain from caffeine, tobacco, and alcohol, for 2 h before the experimental session (Bal et al., 2010). After arrival at the laboratory, participants filled in the BDI (Ghisi et al., 2006) and the State-Trait STAI (Pedrabissi and Santinello, 1989).

**Figure 1:**
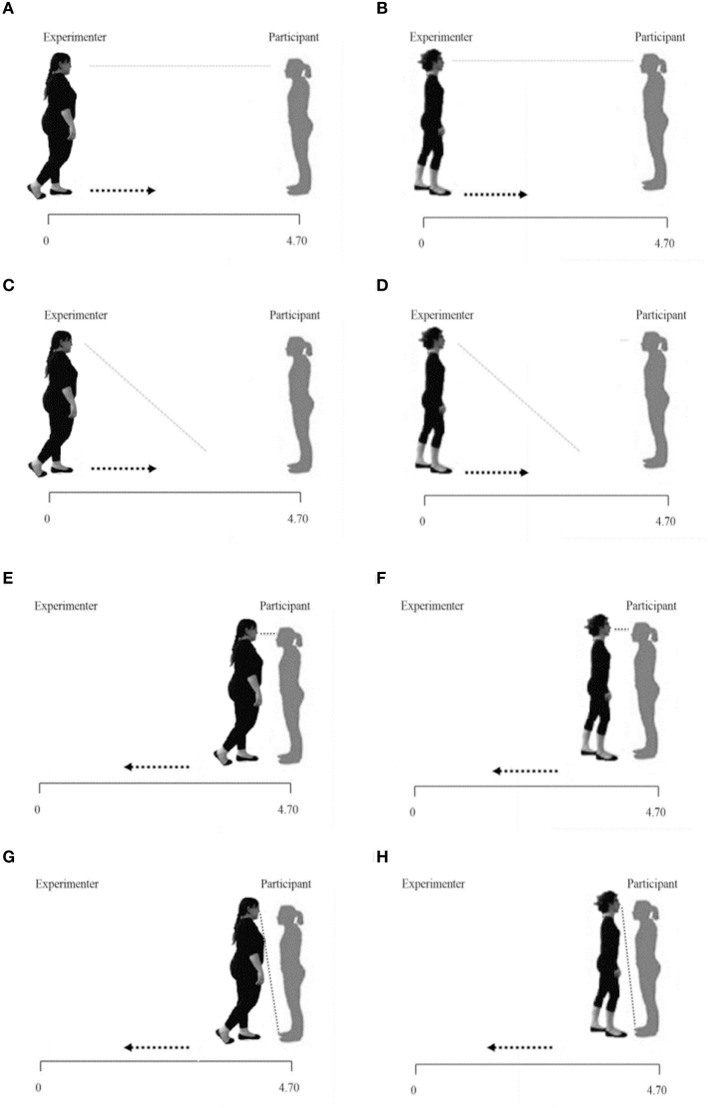
**Physiological and behavioral proxemics task representations**. It show respectively the Fat/Thin Far-gaze conditions **(A,B)**; the Fat/Thin Far-No gaze conditions **(C,D)**; the Fat/Thin Near-gaze conditions **(E,F)**; the Fat/Thin Near-No gaze conditions **(G,H)**.

Both groups of participants performed, in the following order: (1) the Physiological proxemics task; (2) the Heartbeat Perception Task; (3) the Behavioral proxemics task (Kennedy et al., 2009, see below and Figure 1 for a description of the tasks). Participants' ECG was recorded for the entire duration of the Physiological proxemics task and the Heartbeat Perception Task. Furthermore, at the beginning and at the end of the experimental session, and after the Physiological proxemics task, a 2-min resting baseline ECG recording was done, during which participants were instructed to quietly stand up with their shoulders leaning against the wall, and to look at the blue circle in front of them.

Participants were fitted with 10 mm Ø Ag-AgCl pre-gelled disposable electrodes for ECG recording. ECG data were converted and amplified with an eight-channel amplifier (PowerLab8/30; ADInstruments UK) and displayed, stored, and reduced with LabChart 7.3.1 software package (ADInstruments Inc, 2011). All tasks were carried out in the same quiet and softly illuminated room and participants were instructed to relax and remain as still as possible during recording to minimize motion artifacts.

#### Physiological proxemics task

Participants stood up at an end of a 470 cm strip previously placed on the floor, in a comfortable and relaxed position, leaning against the wall.

The experiment consisted in two blocks in which a female experimenter slowly approached or distanced herself from the participant, along the strip, (from 470 to 30 cm, or vice versa, frontally). In the first block, the experimenter had an underweight BMI (Thin condition: 17.5 Kg/m2) and in the second block, the experimenter had an obese BMI (Fat condition: 34 Kg/m2). Both the experimenters were dressed in the same way, wearing a black tracksuit (Figure 1). The order of the two blocks was counterbalanced across participants.

Participants were instructed to pay attention and always follow with their gaze the experimenter and reassured that the experimenter would have never touched them.

Each experimental block consisted of 16 trials (4 for each condition presented in random order). Following audio cues, each experimenter could move along the strip:

- starting from 470 to 30 cm from the participant while looking in the participant's eyes (Far-gaze condition);
- starting from 470 to 30 cm from the participant while glancing down (Far-No gaze condition);
- starting from 30 to 470 cm from the participant while looking in the participant's eyes (Near-gaze condition);
- starting from 30 to 470 cm from the participant while glancing down (Near-No gaze condition).

Each trial lasted 30 s with an inter-trial interval of 15 s.

#### Heartbeat perception task

Heartbeat perception was assessed using the Mental Tracking Method (Schandry, 1981), which is has been widely used in IA evaluation. It highly correlates with other heartbeat detection tasks (Knoll and Hodapp, 1992) and has good test–retest reliability (up to 0.81; Mussgay et al., 1999; Pollatos et al., 2007). Participants were required to start silently counting their own heartbeats, only the heartbeats about which they were sure, on an audio-visual start cue until they received an audio-visual stop cue. The experiment started after one brief familiarization period (15 s) and consisted of four different time intervals (25, 35, 45, and 100 s) presented in random order. Participants were asked to tell a third experimenter the number of heartbeats counted at the end of each interval. During the task, no feedback on the length of the counting phases or the quality of their performance was given and they were not permitted to take the pulse, and Heartbeat perception score, was calculated as the mean score of four separated heartbeat perception intervals according to the following transformation (Schandry, 1981; Pollatos et al., 2008):

$$\begin{array}{l} \frac{1}{4}\sum \left(1-\frac{\left(\left|\text{recorded beats}-\text{ counted beats}\right|\right)}{\text{recorded beats}}\right) \end{array}$$

According to this transformation, heartbeat perception score vary between 0 and 1, with higher scores indicating small differences between recorded and counted heartbeats (i.e., higher interoceptive sensitivity).

#### Behavioral proxemics task

In this third phase, the Physiological proxemics task was repeated but in this case, participants stopped the experimenter at the distance at which they felt most comfortable. Shoulder-to-shoulder distance was recorded using a digital laser measurer. In this phase, the ECG was not recorded.

### ECG recording

Three Ag/AgCl pre-gelled electrodes (ADInstruments, UK) with a contact area of 10 mm diameter were placed on the wrists of the participants in an Einthoven's triangle configuration monitoring ECG (Powerlab and OctalBioAmp 8/30, ADInstruments, UK). The ECG was sampled at 1 KHz and filtered online by the mains filter, which have a negligible distorting effect on ECG waveforms. R-wave peak of the of the ECG was detected from each sequential heartbeat and the R-R interval was timed to the nearest ms. During the editing, a software artifacts detection (artifacts threshold 300 ms) was followed by a visual inspection of the recorded signal. Following standard practices (Berntson et al., 2007) artifacts were then edited by integer division or summation.

The amplitude of RSA was calculated with CMetX (available from http://apsychoserver.psych.arizona.edu). This is a time-domain method but allows derivation of components of heart rate variability within specified frequency bands (Berntson et al., 1997) as spectral techniques. RSA was evaluated as the natural log of variance of heart rate activity across the band of frequencies associated with spontaneous respiration.

RSA estimates were calculated as follows (Allen et al., 2007) (1) linear interpolation at 10 Hz sampling rate; (2) application of a 241-point FIR filter with a 0.12–0.40 Hz band pass; (3) extraction of the band passed variance; (4) transformation of the variance in its natural logarithm.

Coherently with guidelines (Berntson et al., 1997), these procedures were applied to epochs of 30 s, which was the duration of each experimental trial. Then, RSA-values corresponding to Far-gaze, Far- no gaze, Near- gaze, Near No-gaze conditions in each block (Thin or Fat) were separately computed as the average of four 30 s—epochs. Consistently, RSA-values corresponding to baseline and recovery were computed as the average of the four 30 s—epochs. RSA response to Far-gaze, Far- no gaze, Near- gaze, Near No-gaze condition were then separately obtained for the two Thin/Fat blocks as changes from the resting baseline RSA-values to the reactivity during each condition.

For assessing the heartbeat perception score, heart rate data were used.

## Results

### Sample description and questionnaire data

Group comparisons of socio-demographic features (age, BMI) and questionnaire data obtained for the two participant groups were performed with a series of independent samples two tailed *t*-tests, revealing a significantly lower weight and BMI for patients with anorexia nervosa than controls. No differences emerged with respect to height or age. Patients with anorexia nervosa also scored significantly higher in interoceptive awareness deficits (EDI3-ID), depression (BDI), state, and trait anxiety (STAI state and trait), dissociative experiences (DES), general psychopathology (SCL-90 total score), body image concerns (BSQ; BUT, BIC scale), and body image disturbances (BUT, GSI scale). Furthermore, ANg also obtained higher scores in problems with self-esteem (EDI3-LSE), interpersonal insecurity (EDI3-II), and emotive dysregulation (EDI3-ED; see Table 1).

### Between-groups differences in social disposition at rest, and its relations with psychological variables

To assess the presence of significant differences between ANg and HCg in social disposition at rest, we carried out a repeated measures ANCOVA with Group (ANg vs. HCg) as between factor and Condition (baseline vs. recovery) as within factor. In addition, since the previously found difference in terms of resting RSA between and within the two groups could be influenced by age, BMI, anxiety, and depression, age, BMI, scores obtained from STAI Trait, STAI State, and BDI questionnaires were added to the model as covariates. The factor Condition was introduced because it is well-known that in situations demanding sustained attention, or with challenging stimuli, RSA is suppressed (Porges, 1995). Therefore, we contrasted Baseline and Recovery in each Group to disentangle this possible confounding effect on our results. For this analysis, we excluded a participant in the control group because we did not entirely recorded RSA for technical problems.

**Figure 2:**
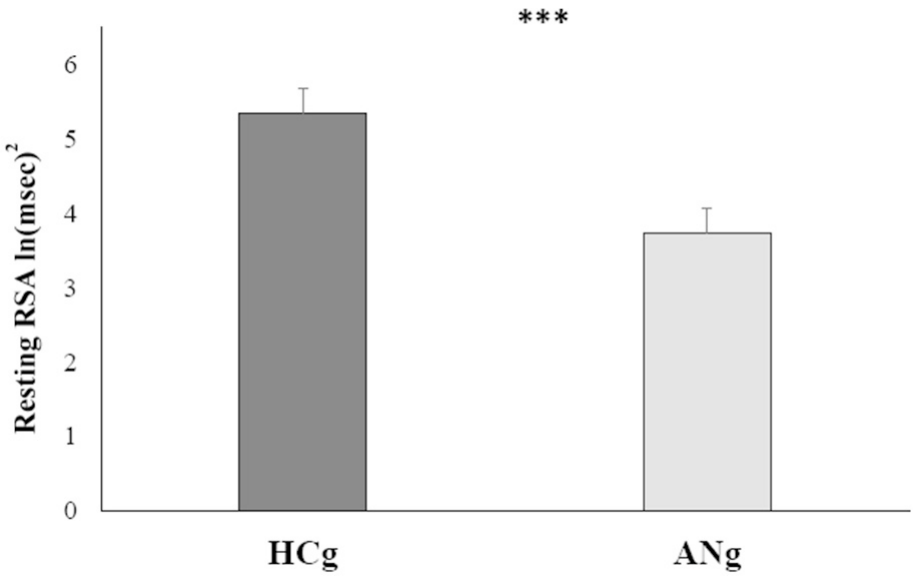
**Marginal means of resting RSA levels for both HCg and ANg**. Covariates included in the model were estimated to the following values: age = 22.8, BMI = 18.46, STAI-Trait = 51.63, STAI-State = 42.98, BDI = 17.31. Error bars depict the standard error of the mean; ^***^p < 0.001.

The repeated measures ANCOVA only revealed the main effect of Group [*F*_(1, 40)_ = 7.6; *p* > 0.01, η^2^ = 0.16; ANg: mean = 3.7 ln (ms)^2^, SE = 0.32; HCg: 5.3 ln (ms)^2^, SE = 0.33] even controlling for age, BMI, STAI-Trait, Stai-State, and BDI. Except age [*F*_(1, 40)_ = 8.7, *p* < 0.01, η^2^ = 0.18], none of these covariates resulted significant [BMI: *F*_(1, 40)_ = 0.29, *p* > 0.6, η^2^ = 0.007; STAI-Trait score: *F*_(1, 40)_ = 2, *p* > 0.1, η^2^ = 0.05; STAI-State score: *F*_(1, 40)_ < 0.001, *p* > 0.9, η^2^ < 001; BDI score: *F*_(1, 40)_ = 1.15, *p* > 0.2, η^2^ = 0.03] (see Figure 2).

Additionally, neither the main effect of Condition [*F*_(1, 40)_ = 3.8 *p* > 0.4; η^2^ = 0.01; ANg: marginal mean = 4.4 ln (ms)^2^, SE = 0.17; HCg: marginal mean 4.7 ln (ms)^2^, SE = 0.15] nor the interaction with Group were significant [*F*_(1, 40)_ = 0.008; *p* > 9; η^2^ < 0.001], showing that after controlling for the inserted covariates, RSA responses in baseline (ANg: marginal mean = 3.8, SE = 0.32; HCg: marginal mean = 5.5, SE = 0.33) and recovery conditions (ANg: marginal mean = 3.6, SE = 0.36; HCg: marginal mean = 5.2, SE = 0.38) did not differ within each group.

To assess the possible influence of the different age of onset and duration of the illness for each AN patient (see Table 2) on her resting RSA we conducted two separate correlations. The first correlation was performed between Resting RSA and Age of Onset, the second between resting RSA and Duration of the Illness (in years). Since the variables “Duration of Illness” and “Age of Onset” were not distributed normally (Age of Onset: Shapiro-Wilk test *p* < 0.05, Duration of Illness: Shapiro-Wilk test, *p* < 0.001)] we chose non-parametric Spearman correlations.

**Table 2:** **Information relative to age, age of onset, and duration of the illness, IA, and resting RSA of ANg**.

**ID**	**Age**	**Age of the onset**	**Duration of the illness (year)**	**IA**	**Resting RSA ln (ms)^2^**
1	22	19	3	0.36	5.08
2	13	12	1	0.30	3.86
3	19	17	2	0.51	2.80
4	39	18	21	0.71	3.23
5	44	17	27	0.41	2.15
6	31	15	16	0.25	2.39
7	15	15	0	0.53	1.79
8	17	16	1	0.35	4.03
9	32	17	15	0.30	3.67
10	16	15	1	0.33	5.93
12	23	15	8	0.36	6.14
13	22	14	8	0.66	2.65
14	23	16	7	0.69	5.10
15	17	14	3	0.37	3.48
16	21	20	1	0.67	3.61
17	21	21	0	0.36	3.82
18	27	24	3	0.55	2.03
19	48	24	24	0.73	1.55
20	24	19	5	0.34	5.06
21	15	14	1	0.59	2.76
22	16	14	2	0.84	3.07
23	17	16	1	0.37	4.19
24	16	15	1	0.57	4.90
25	15	14	1	0.35	6.25

Bonferroni-corrected (*p* < 0.025) correlation analysis excluded the significant relationship between resting RSA and Age of Onset (*r*_s_ = −0.16; *p* = 0.23). In addition, neither the relationship between resting RSA and duration of illness was significant (*r*_s_ = 0.27; *p* = 0.103).

### Between-groups differences in IA, and its relations with psychological variables

Since previous data indicated that IA could be influenced by age, BMI, anxiety and depression (see Introduction), between-groups difference in IA were assesses by and ANCOVA, controlling for age, BMI, and the scores obtained from STAI-Trait and STAI -State and BDI that entered as covariates. The factor Group (ANg vs. HCg) was included as between-factor. Results did not show any significant difference in IA between the two groups [*F*_(1, 42)_ = 0.82; *p* > 0.3, η^2^ = 0.02] since ANg did not show a lower heartbeat perception score compared to HCg (ANg: mean = 0.53 SE = 0.07, HCg: mean = 0.41 SE = 0.07). None of the covariates included in the model resulted significant [age: *F*_(1, 42)_ = 0.36, *p* > 0.5, η^2^ = 0.001; BMI: *F*_(1, 42)_ = 0.9, *p* > 0.3, η^2^ = 0.02; STAI-Trait score: *F*_(1, 42)_ = 0.76, *p* > 0.3, η^2^ = 0.02; STAI-State score: *F*_(1, 42)_ = 0.68, *p* > 0.4, η^2^ = 0.02; BDI score: *F*_(1, 42)_ = 3.27, *p* > 0.05, η^2^ = 0.07].

To evaluate the possible influence of the different age of onset and duration of the illness (see Table 2) on patients' IA, we conducted two separate non-parametric Spearman correlations. The first correlation was performed between IA and Age of Onset, the second, between IA and Duration of the Illness (in years).

Bonferroni-corrected (*p* < 0.025) correlation analysis showed that the variables IA and Age of Onset were not significantly correlated (*r*_s_ = 0.14; *p* = 0.25) as well as the variables IA and Duration of Illness (*r*_s_ = 0.115; *p* = 0.3).

### Relationship between IA and social disposition

To better understand the relationship between IA and social disposition in AN, we carried out a Pearson correlation between IA and resting RSA (mean score between baseline and recovery). To compare the correlation coefficients of the different groups, we used the Fisher r to z transformation (Lowry, 2004; Cohen et al., 2013; Eid and Lischetzke, 2013). For this analysis, we excluded a participant in the HCg because she resulted as a multivariate outlier, with unusual combination of scores on the considered variables.

**Figure 3:**
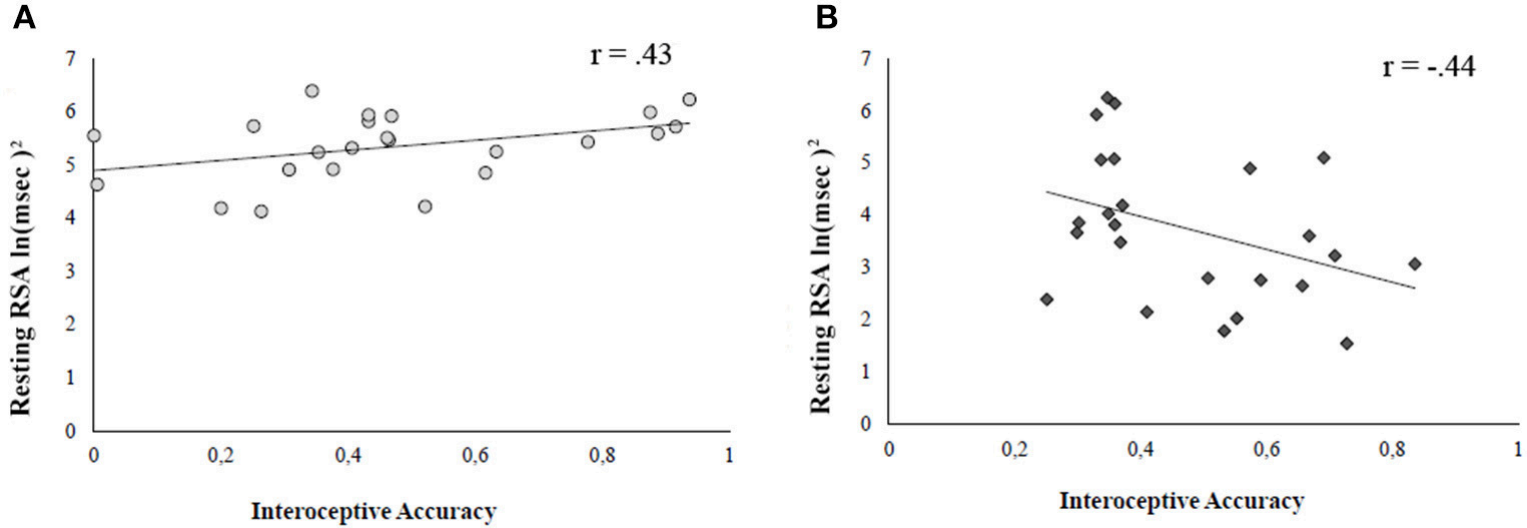
**Pearson correlations between IA and resting RSA for both HCg (A)** and ANg **(B)**.

The analysis showed a significant relationship between IA and RSA at rest in the two groups, but positive in HCg (*r*_21_ = 0.40; *p* = 0.03) and negative in AN (*r*_22_ = −0.39; *p* = 0.03; *z* = 2.67, *p* = 0.008). This result suggests that, even if we did not find significant differences in IA between HCg and ANg, there is a different association between the two variables in the individuals affected by AN (see Figure 3).

### Physiological proxemics task

To assess changes in autonomic reactivity both in ANg and HCg during social interactions, participants' RSA responses entered in a repeated measures ANOVA with experimenter's BMI (Fat vs. Thin), Distance (far vs. near) and Gaze (gaze vs. no gaze) as within factors and Group (ANg vs. HCg) as between factor. The Tukey test was used for all *post-hoc* comparisons.

**Figure 4:**
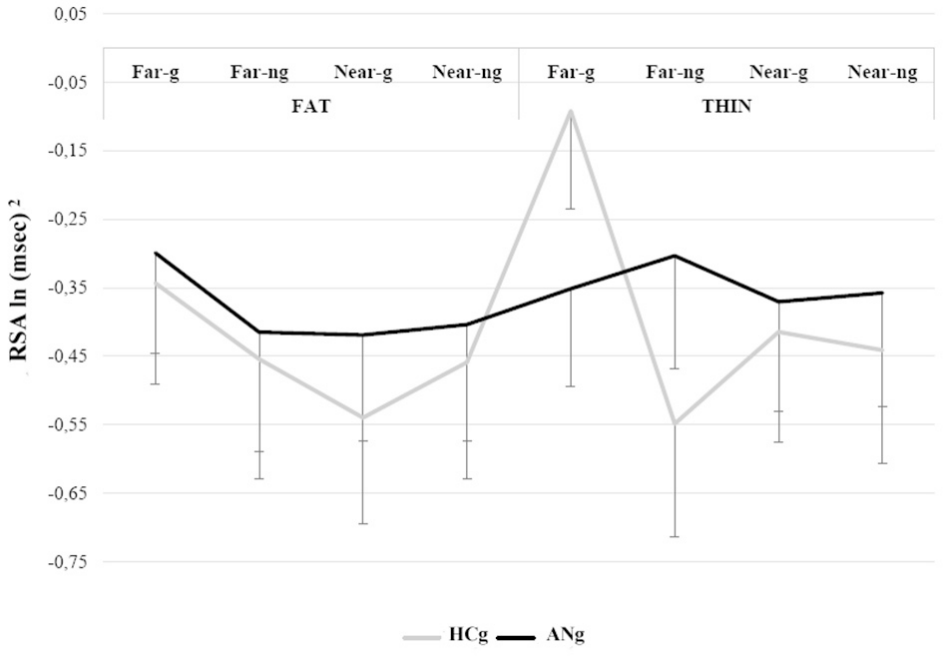
**RSA responses during the Physiological proxemics task of HCg and ANg**. Error bars depict the standard error of the mean; g, gaze; ng, no gaze.

The most relevant significant result (overall results of the ANOVA are reported in Table 3) was the interaction among BMI, Distance, Gaze and Group [*F*_(1, 46)_ = 5.11; *p* < 0.05, η^2^ = 0.10], because of the greater RSA responses for the Thin-Far-Gaze condition than all other conditions [mean = −0.11 ln (ms)^2^; SE = 0.14; all *ps* < 0.05]. This modulation across the experimental conditions was present only for HCg (see Figure 4).

**Table 3:** **ANOVA significant effects of the Physiological proxemics task on RSA responses of ANg and HCg**.

**Effect**	***F*(*df* = 1,46)**	***p***	**η^2^**	**Mean [ln(ms)2](*SE*)**
Distance	6.38	<0.05	0.12	Far = −0.35 (0.10) vs. near = −43 (0.11)
Distance^*^Gaze	8.59	<0.01	0.16	Far eye = −0.27 (0.10) vs. far no eye = −0.43 (0.11), near eye = −0.44 (0.10), near no eye = −0.41 (0.11); all *ps* < 0.01
Distance^*^Gaze^*^Group	4.6	<0.05	0.09	**HCg**. Higher RSA responses in far eye = −22 (0.13); vs. far no eye = −50 (0.16), near eye = −0.48 (0.15), near no eye = −0.45 (0.16); all *ps* < 0.01.
				ANg. No differences among conditions: far eye = −0.32 (0.13), far no eye = −0.36 (0.16), near eye = −0.39 (0.15), near no eye = −0.38 (0.16); all *ps* = n.s.
BMI^*^Gaze^*^Group	6.57	<0.05	0.12	**HCg**. Higher RSA responses in thin eye = −0.25 (0.16) vs. thin no eye = −0.49 (0.17), fat eye = −0.44 (0.15), fat no eye = −0.46 (0.17); all *p*_*s*_ < 0.05.
				ANg. No differences among conditions: thin Eye = −0.36 (0.15) vs. thin no eye = −0.33 (0.16), fat eye = −36 (0.15), fat no eye = −0.41(0.15); all *p*_*s*_ n.s.
BMI^*^Distance^*^Gaze^*^Group	5.11	<0.05	0.10	**HCg**. Greater RSA responses in the thin far−eye condition = −0.1 (0.14) than all other conditions: thin far−no eye = −0.55 (16), thin near eye = −0.41 (16), thin near−no gaze = −0.44 (0.17), fat far eye = −0.34 (0.15), fat far− no-eye = −0.45 (0.17), fat near eye = −54 (0.15), fat near-no eye = −0.46 (0.17); all *ps* < 0.05.
				ANg. No differences among conditions: fat far eye = −0.30 (0.15), fat far –no eye = −0.41 (0.17), fat near eye = −0.42 (0.15), fat near-no eye = −0.40 (0.17), thin far eye = −0.35 (0.14), thin far-no eye = −0.30 (0.16), thin near eye = −0.37 (0.16), thin near-no eye = −0.35 (0.17); all *ps* n.s.

n.s, not significant;

* *= interacting*.

### Behavioral proxemics task

To assess changes in behavioral responses both in ANg and HCg during social interactions and to explore possible differences in autonomic reactivity between the two groups, participants' rating of comfort (reciprocal normalized data; Barbaranelli and D'Olimpio, 2006) entered in a repeated measures ANOVA. Experimenter's BMI (Fat vs. Thin), Distance (far vs. near) and Gaze (gaze vs. no gaze) were inserted as within factors, and Group (ANg vs. HCg) as between factor. We used the Tukey test for all *post-hoc* comparisons.

**Figure 5:**
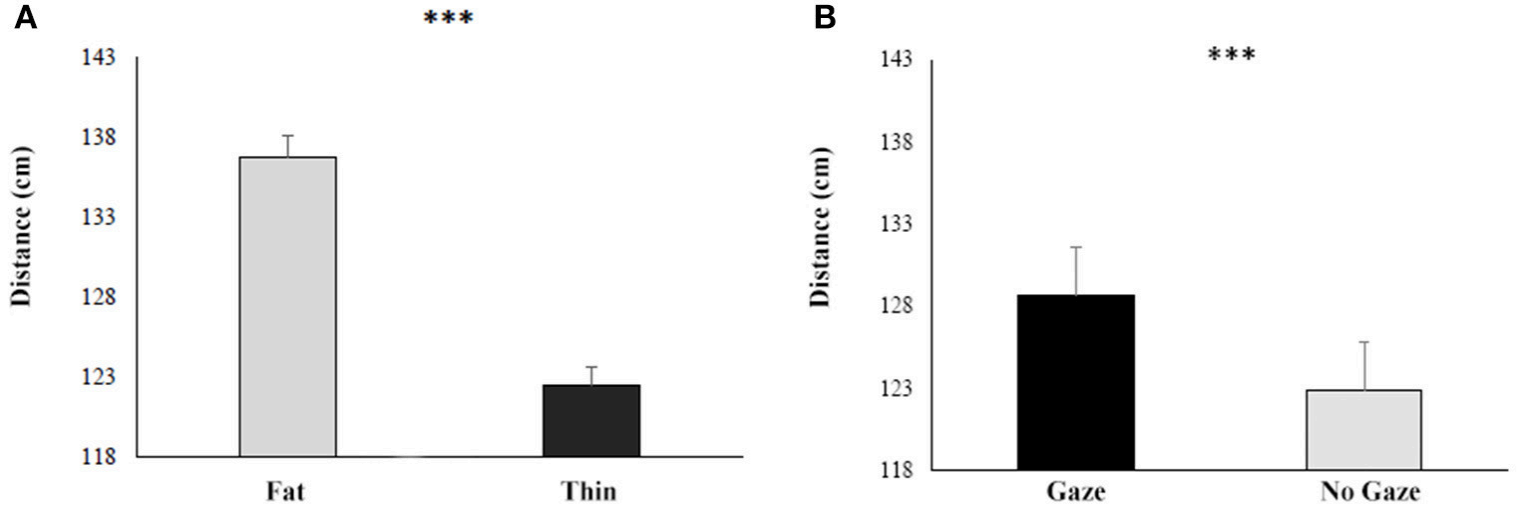
**Responses during the Behavioral proxemics task of both HCg and ANg in function of the BMI (A)** and the Gaze **(B)**. Error bars depict the standard error of the mean. ^***^p > 0.001.

The ANOVA showed that (overall results of the ANOVA are reported in Table 4) the main factors BMI [*F*_(1, 46)_ = 40.2; *p* < 0.001; η^2^ = 0.47] was significant: both groups, indeed, felt comfortable with the thin experimenter, stopping her 13 cm closer than the fat one (Fat: mean = 132 cm SE = 0.06 vs. Thin: mean = 119 cm, SE = 0.06; see Figure 5A).

**Table 4:** **ANOVA significant effects of the Behavioral proxemics task on RSA responses of ANg and HCg**.

**Effect**	**F(*df* = 1,46)**	***p***	**η^2^**	**Mean (cm) (SE)**
BMI	40.2	<0.001	0.47	Fat = 132 (0.06) vs. thin = 119 (0.11)
Distance	24.2	<0.001	0.34	Far = 118 (0.06) vs. near = 133 (0.07)
Gaze	7	<0.05	0.13	Gaze = 129 (0.07), no gaze = 123 (0.06)
BMI^*^Distance	15.6	<0.001	0.25	Fat far = 119 (0.13) vs. fat near = 145 (0.07); *p* < 0.01; thin far = 116 (0.07), thin near = 122 (0.06); *p* > 0.05
Distance^*^Gaze	8.4	<0.01	0.15	Participants stopped closer the experimenter in the far condition, approaching them and glancing down than all other condition (all ps < 0.001): far-gaze = 122 (0.07); far-no gaze = mean = 113 (0.06), near gaze: = 135 (0.07); near-no gaze = 133 (0.06).
Distance^*^Group	4.1	<0.05	0.08	**HCg**. Differentiate between the starting distance of the experimenter; far = 103 (0.08) vs. near = 127 (0.09); *p* = < 0.001. **ANg**. On the contrary, did not differentiate between far = 131 (0.08) and near = 141 (0.1) *p* = n.s.

* *Interacting; n.s, not significant*.

The interaction between BMI and Distance was significant [*F*_(1, 46)_ = 15.6; *p* < 0.001; η^2^ = 0.25), since participants felt more comfortable with both the thin and fat experimenter in the Far condition than in the Near condition [(Fat-Far: mean = 119 cm, SE = 0.06; Fat-Near: mean = 145 cm SE = 0.07; *p* < 0.01); (Thin-Far: mean = 116 cm, SE = 0.07; Thin-Near: mean = 122 cm SE = 0.06; *p* < 0.001)].

Also the factor Gaze resulted significant [*F*_(1, 46)_ = 7; *p* < 0.05; η^2^ = 0.13], showing that both groups stopped the experimenter 7 cm closer when she was glancing down than when the experimenter maintained the eye contact with participants (Gaze: mean = 129 SE = 0.07 vs. No Gaze: mean = 123 SE = 0.06; see Figure 5B).

### Relations between social disposition at rest and tolerance of social distances

**Figure 6:**
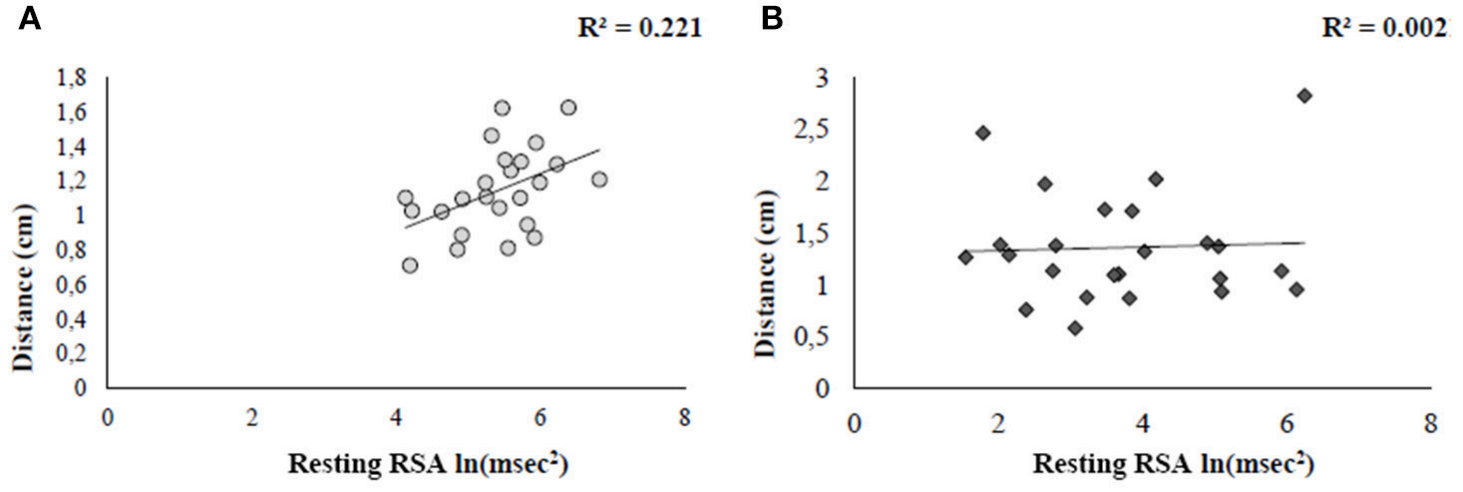
**Linear regression plots showing the relation between comfort ratings and social disposition (resting RSA) for both HCg (A)** and ANg **(B)**.

In order to investigate the role of autonomic arousal in guiding behavioral responses in social distances, two linear regression analyses, having Distance as criterion (calculated as the overall mean among participants' rating of comfort) and RSA at rest as predictor were independently performed for the two groups. Results revealed a significance relationship in HCg (*t* = −2.5, *b* = −0.47, *p* < 0.05) explaining the 22% of the variance [*F*_(1, 22)_ = 6.3, *p* < 0.05, *R*^2^ = 0.22, *R*^2^ adjusted = 0.19, see Figure 6A). When the same regression was conducted on ANg, the regression model was not significant (*t* = 0.45, *b* = −0.1, *p* > 0.6), explaining only the 0.2%) of the variance [*F*_(1, 21)_ = 0.20, *p* > 0.6, *R*^2^ = 0.002; *R*^2^ adjusted = −0.04, see Figure 6B).

## Discussion

Basing on the idea that the ability to adapt oneself to the social settings does not depend only from high sensitivity in assessing information from the outer environment, but also from the inner body (Paladino et al., 2010; Tsakiris et al., 2011; Tajadura-Jiménez et al., 2012; Gaudio et al., 2014), the present study aimed to explore both the relationship between autonomic functioning and IA, in a population of restrictive anorexic patients whose interoception is impaired (Fassino et al., 2004; Friederich et al., 2006; Nunn et al., 2008; Pollatos et al., 2008; Paladino et al., 2010; Strigo et al., 2013; Gaudio et al., 2014). Furthermore, the autonomic reactivity of ANg during social interactions and their behavioral judgment of social distances was explored, manipulating social cues and social desirability traits of experimenters (BMI) interacting with participants.

To these purposes, we recorded RSA responses of both HCg and ANg during both a resting condition and social interaction (Physiological proxemics task). Then we submitted both groups to a well-assessed heartbeat perception task (Schandry, 1981). Finally, to better interpret our results, we submitted participants to an “overt” behavioral version of the Physiological proxemics task.

In line with our hypothesis, we found that ANg showed lower RSA at rest than HCg. Since higher resting RSA is an index of self-regulation, social disposition, and it is considered a marker of positive social functioning in autism (Bal et al., 2010; Patriquin et al., 2013), our results revealed a lower social disposition in ANg.

Concerning IA, contrary to Pollatos et al. (2008), in the heartbeat detection task ANg did not perform differently from HCg. It should be emphasized that even our HCg showed lower IA compared to the literature (Pollatos et al., 2008; Ainley et al., 2012; Herbert et al., 2012; Klabunde et al., 2013; Krautwurst et al., 2014). An explanation of this result may be that the difficulty of this task lead to higher level of stress and arousal in our sample. As recently showed by Khalsa et al. (2015), AN and HC did not differ in the detection of interoceptive changes occurring during isoprotenol infusion in situations of high arousal (see Khalsa et al., 2015 for full details). Our results do not necessarily entail that IA is not compromised in anorexic patients, or that IA might not have a role in AN disturbances (e.g., Herbert and Pollatos, 2012; Eshkevari et al., 2014). The most interesting and novel result of our study is the different association between IA and RSA between ANg and HCg. In fact, even if we did not found a reduced IA in ANg, as espected we found that higher IA is related to higher social disposition (higher RSA at rest) in HCg, but not in ANg, in which these two variables are disfunctionally associated.

In the Physiological proxemics task, ANg showed flattened autonomic reactivity across all experimental conditions. Anorexic patients seemed not to be engaged in social interactions; they did not respond differently to the presence of two different experimenters, and to the manipulation of significant social cues, such as the eye contact (Argyle and Dean, 1965; for a review see Kleinke, 1986) and the body size of the experimenter. On the contrary, HCg showed better autonomic reactivity to social stimuli, showing higher RSA responses when the underweight experimenter approached them keeping the eye contact, which has a crucial role for the relevance and intention of social stimuli (Carlston, 2013).

It is possible, however, that the higher RSA in the social task could also reflect effortful emotion regulation in presence of a moderately stressful stimulus (Porges, 2007) caused by social anxiety or by unpleasantness. We can exclude this interpretation of our results for the following reasons: first, regression analysis revealed that participants' anxiety did not significantly contribute to the association between IA and RSA responses; second, the Behavioral proxemics task showed that both ANg and HCg felt more comfortable when interacting with the thin experimenter than with the fat one.

In the overt judgment of comfort in defining social distances, both ANg and HCg felt more comfortable (i.e., stopped the experimenters at closer distance) when the experimenters were glancing down. This results is coherent with several studies suggesting that while direct glance is affiliative, without eye contact we do not feel that we are fully in communication with others (e.g., Argyle and Dean, 1965; Wieser et al., 2009). However, during social interactions, people look at each other frequently, most when they are listening to each other, but for short periods of time (about -10 s). When glances are longer than this, anxiety is aroused (Argyle and Dean, 1965).

A further result of this task concerns the fact that both ANg and HCg (only the latter also showed coherent autonomic responses), felt less comfortable with the obese experimenter, stopping her at longer distances than the thin experimenter. We speculate that these results could reflect the internalization of cultural beliefs related to obese individuals, who are perceived to be less attractive than their thinner counterparts (Harris, 1990; Sobal et al., 2006; Puhl and Heuer, 2009). A recent study indeed showed that medical students' level of visual contact with their patient differed depending on the patient's weight (Persky and Eccleston, 2011). This result is in line with the “objectification theory” suggested by Fredrickson and Roberts (1997) stating that every culture has a shared concept of ideal beauty that is internalized by individuals (especially by women) whose satisfaction or dissatisfaction depends on to what extent they meet such a standard. Self-objectification is more pervasive in eating disorders (e.g., Calogero et al., 2005) and is also inversely related to interoception (Myers and Crowther, 2008).

The last point to be addressed is the relationship between resting RSA and the comfort rating of social distances. While we found a clear positive association between these two variables in HCg, this relation was lacking in ANg. In other words, the higher is the autonomic social disposition in HCg, the wider is their tolerated proxemics distance, suggesting that the higher is the social disposition, the wider is the distance at which they are socially engaged/efficient. The lack of this relation in ANg finds support in studies showing lack of emotional clarity in ANg (Damasio, 2004; Merwin et al., 2010). Emotional clarity is conceptualized as the clarity regarding one's internal experiences/arousal (Merwin et al., 2010), which is nothing but another way to define interoception.

Taken together, our results suggests that ANg, contrarily to HCg, are affected both by lower social disposition and more flattened autonomic reactivity in social context, irrespective of social cues and body size of the interacting experimenters. Moreover, while in HCg the autonomic functioning supports the behavioral regulation of social distances, this is not true for ANg whose altered autonomic functioning is not only abnormally related to interoceptive accuracy, but also coherently correlates with lack of emotional clarity and abnormal conditioned responses such as binging, purging, fasting, and other compensatory behaviors (Brogan et al., 2010). These findings support an embodied view of this illness, emphasizing that AN might be a more pervasive disorder involving, beyond mere cognitive factors, a sort of “flattened sense of the physical body,” which may contribute to reinforcing AN symptoms and generate altered meanings, emotions and social behaviors. These results can be also predicted by the etiological model of Riva (2014; see Introduction), which suggests that eating disorders, in the course of the evolution of their bodily experience integrating the manifold levels of bodily representation over time, may be locked in the “objectified body” (Riva, 2014), that is, in an allocentric perspective in which the body is experienced as an object, disconnected and not updated by multisensory perception, which normally contribute to the egocentric view of the body (Riva and Gaudio, 2012).

Possible limitations of this study are the small sample of participants and the fact that the HCg a was restricted to students. We also did not introduce measures for alexithymia, disgust propensity and sensitivity, which seem to be related to interoception and have some implications for social cognition. However, to the best of our knowledge, this is the first study exploring the autonomic correlates of social contexts in eating disorder and its link with the ability to perceive the inside of the body. Even if further studies are necessary to formulate a complete etiologic model of this illness, we suggest that future treatments should take into account the altered bodily correlates of self-experience and their neurobiological dysfunctions. That would allow the development of more effective strategies able to reduce treatment resistance, a frequent issue in eating disorders (Kaye et al., 2009; Treasure and Schmidt, 2013).

## Author contributions

MAm designed the study, collected, analyzed, and interpreted the data, she wrote the manuscript. MAr was involved in study design, collection of data and analyses. She also contributed to the drafting of the manuscript. ER and Fd were principally engaged in the recruitment of participants and data collection, furthermore they contributed to results interpretation. MS, PV, PT, and SM were involved in the recruitment of participants and data collection and took part to the results interpretation. VG designed the study, interpreted the data and drafted the manuscript. All the authors approved the final version of the manuscript.

## Funding

This research was supported by a grant of Chiesi Foundation and by the Einstein Stiftung Fellowship to VG.

### Conflict of interest statement

The authors declare that the research was conducted in the absence of any commercial or financial relationships that could be construed as a potential conflict of interest. The reviewer GR and handling Editor declared their shared affiliation, and the handling Editor states that the process nevertheless met the standards of a fair and objective review.
